# Increased Markers of Oxidative Stress and Positive Correlation Low-Grade Inflammation with Positive Symptoms in the First Episode of Schizophrenia in Drug-Naïve Patients

**DOI:** 10.3390/jcm11092551

**Published:** 2022-05-02

**Authors:** Ewa Dudzińska, Kinga Szymona, Jacek Bogucki, Wojciech Koch, Ewelina Cholewińska, Robert Sitarz, Katarzyna Ognik

**Affiliations:** 1 Department of Food and Nutrition, Medical University of Lublin, 20-093 Lublin, Poland; wojciechkoch@umlub.pl; 2 Department of Psychiatry, Psychotherapy and Early Intervention, Medical University of Lublin, 20-439 Lublin, Poland; szymona.kinga@gmail.com; 3 Chair and Department of Organic Chemistry, Medical University of Lublin, 20-093 Lublin, Poland; jacekbogucki@umlub.pl; 4 Department of Biochemistry and Toxicology, University of Life Sciences in Lublin, 20-950 Lublin, Poland; ewelina.cholewinska@up.lublin.pl (E.C.); katarzyna.ognik@up.lublin.pl (K.O.); 5 Department of Human Anatomy, Medical University of Lublin, 20-090 Lublin, Poland; r.sitarz@umlub.pl; 6 Department of Surgical Oncology, St. John’s Cancer Center, 20-090 Lublin, Poland

**Keywords:** schizophrenia, oxidative stress, low-grade inflammation

## Abstract

Schizophrenia is a severe and chronic mental illness usually diagnosed in adolescents and young adults. Many studies indicate that oxidative stress causes membrane dysfunction and cell damage, which is implicated in the pathophysiology of schizophrenia. The purpose of our study was to evaluate oxidative stress markers (the main primary products of lipid peroxidation, lipid hydroperoxides (LOOH), and end products of lipid peroxidation, malondialdehyde (MDA), superoxide dismutase (SOD), glutathione (GSH), and Ferric Reducing Ability of Plasma (FRAP)) in the plasma of patients with the first episode of schizophrenia in drug-naïve patients (22 men and 12 women aged 17–29). The control group (Ctrl) comprised 26 healthy subjects (19 men and 7 women, aged 18–30 years). The Positive and Negative Syndrome Scale (PANSS) was applied to evaluate psychotic symptoms. Analyses of the oxidative stress variables revealed an increased level of SOD (U/mL) in subjects with schizophrenia versus control group. In addition, lipid damage measured as LOOHs µ (mol/L) and MDA was significantly higher in patients with schizophrenia in comparison to control subjects. There was a positive correlation between MDA µmol/L and PANSS P and a positive correlation between C-reactive protein (CRP) and the PANSS P scale. The elevated level of superoxide dismutase in patients with the first episode of schizophrenia can be explained by compensatory mechanisms to counteract oxidative stress. Malondialdehyde can be used as a simple biomarker of low-grade systemic inflammation associated with oxidative stress. A positive correlation between CRP and PANSS P scale and MDA and PANSS P scale may indicate a significant relationship between the development of low-grade inflammation and damage associated with oxidative stress in the development of the first symptoms of schizophrenia.

## 1. Introduction

Schizophrenia is a severe and chronic mental illness usually diagnosed in adolescents and young adults [1]. Despite the large number of studies, the precise cause of schizophrenia is still unknown. The illness has various phenotypes determined by a number of factors, which may be genetic [2] or non-genetic, such as prenatal and neonatal infections, birth complications, famines, maternal malnutrition, drug and alcohol abuse, and lifestyle [3]. Some of the studies show that the activation of the maternal immune system associated with redox imbalance is one of the most important risk factors for the development of schizophrenia [4]. These can be both pro-inflammatory factors related to the infection as well as factors related to experiencing maternal stress during pregnancy or prenatal malnutrition. It has been shown that low protein intake during pregnancy induces mitochondrial dysfunction and a decrease in endogenous antioxidants [4]. In all cases, there is the release of pro-inflammatory cytokines and the production of reactive oxygen species (ROS) [4].

The brain is preferentially susceptible to oxidative damage since it is under very high oxygen tension and highly enriched in ROS susceptible proteins, lipids, and poor DNA repair [3]. Oxidative stress leads to oxidative cell damage, causing DNA breaks and protein inactivation, resulting in altered gene expression and peroxidative loss of membrane phospholipids causing abnormal cell growth and differentiation and, as a consequence, apoptotic cell death [3]. 

A balance between ROS production and utilization is important to maintain ROS-dependent cellular processes and prevent ROS-induced cell damage [4].

The enzymatic antioxidant defense system of the human body includes superoxide dismutase (SOD), catalase (CAT), and glutathione peroxidase (GpX), which prevent the initiation of chain reactions of reactive species [5]. Dopamine, which has neurotoxic potential, has been shown to contribute to cellular oxidative stress, which may increase in the case of the impaired synthesis of antioxidants [6].

High levels of reactive oxygen species or other free radicals may directly damage lipids. The primary lipid peroxidation products are lipid hydroperoxides (LOOH). Secondary products of lipid peroxidation include many aldehydes, among which malondialdehyde (MDA) seems to be the most mutagenic [7].

Much research indicates that oxidative stress causes membrane dysfunction and cell damage, which are implicated in the pathophysiology of schizophrenia [2]. 

Antipsychotic drugs are a standard treatment for schizophrenia. The mechanism of action of antipsychotic medications and their potential effects on cellular processes in the brain may have a neuroprotective function in schizophrenia. Antipsychotic medications can be divided into the first generation (typical, e.g., haloperidol) and the second generation (atypical, e.g., risperidone, clozapine, olanzapine, aripiprazole, quetiapine, and ziprasidone) [8]. 

Some studies have shown that oxidative stress may be induced by first-generation antipsychotics. On the other hand, atypical antipsychotic drugs may lessen oxidative damage, improve plasma total antioxidant status, and reduce lipid peroxidation damage [9].

Therefore, the aim of our study was to assess oxidative stress markers (the main primary products of lipid peroxidation, lipid hydroperoxides (LOOH), and end products of lipid peroxidation, malondialdehyde (MDA), superoxide dismutase (SOD), glutathione (GSH), and Ferric Reducing Ability of Plasma (FRAP)) in the plasma of patients with the first episode of schizophrenia in drug-naïve patients.

The conducted studies will respond to the question of whether the development of schizophrenia is associated with an increased level of oxidative stress and whether there is an ROS relationship with an increase in inflammatory markers, such as the CRP reactive protein (CRP).

## 2. Materials and Methods

We measured the levels of oxidative stress markers in plasma of 34 schizophrenia drug-naïve patients (22 men and 12 women aged 17–29) with their first schizophrenia episode, diagnosed in accordance with ICD-10 criteria (F20). The study subjects were hospitalized at the I Department of Psychiatry, Psychotherapy, and Early Intervention in Lublin or were outpatients of the Hospital Mental Health Clinic at the Children’s University Hospital in Lublin. The Positive and Negative Syndrome Scale (PANSS) was applied to evaluate psychotic symptoms.

The control group (Ctrl) comprised 26 healthy subjects (19 men and 7 women, aged 18–30 years), matched for gender and age, with no family history of mental illness. The exclusion criteria for the study were any of the following: inflammatory, neurological, or autoimmune diseases (active or past); dependence on alcohol or other addictive substances; and intellectual impairment determined by psychiatric examination. Moreover, additional factors of exclusion from the study, as in the group of patients with schizophrenia, were incorrect BMI and smoking.

Blood samples were collected in EDTA-containing tubes immediately after enrollment and processed following standard procedures. The blood samples of the centrifuged plasma were separated and immediately frozen and stored at −80 °C until analysis. All samples were analyzed in a single batch.

Oxidative stress was evaluated by measuring the activity of superoxide dismutase (SOD) and catalase (CAT), content of the products of lipid peroxidation, such as lipid hydroperoxides (LOOH) and malondialdehyde (MDA), as well as the total antioxidant potential (FRAP) in the plasma, which was determined according to methods described by Ognik and Wertelecki (2012) [10].

### Statistical Analyses

The distribution of the variables was checked with the usage of analysis of histograms and the Shapiro–Wilk test in Statistica software (Statistica v.13, Polish version from StatSoft Corporation Poland, the partner of Tibco Corporation, Palo Alto, CA, USA.) The statistical analysis of the results obtained in this study included descriptive statistics, nonparametric tests (since data distribution deviated from the normal distribution), U Mann–Whitney, and r Spearman coefficient.

## 3. Results

The first step in the data analysis was to compute descriptive statistics for oxidative stress variables. The results of the analysis are presented in Table 1.

The next stage of data analysis was to check whether the values of the analyzed parameters in the group of patients with schizophrenia differed statistically significantly compared to the control group. The results of the analysis are presented in Table 2.

Analyses of the oxidative stress variables revealed an increased level of SOD in subjects with schizophrenia versus control group. In addition, lipid damage (measured as LOOHs and MDA) was significantly higher in patients with schizophrenia in comparison to control subjects. The other differences between CAT and FRAP were not statistically significant. 

Figures showing statistically significant differences in the levels of oxidative stress variables are presented below.

**Figure 1:**
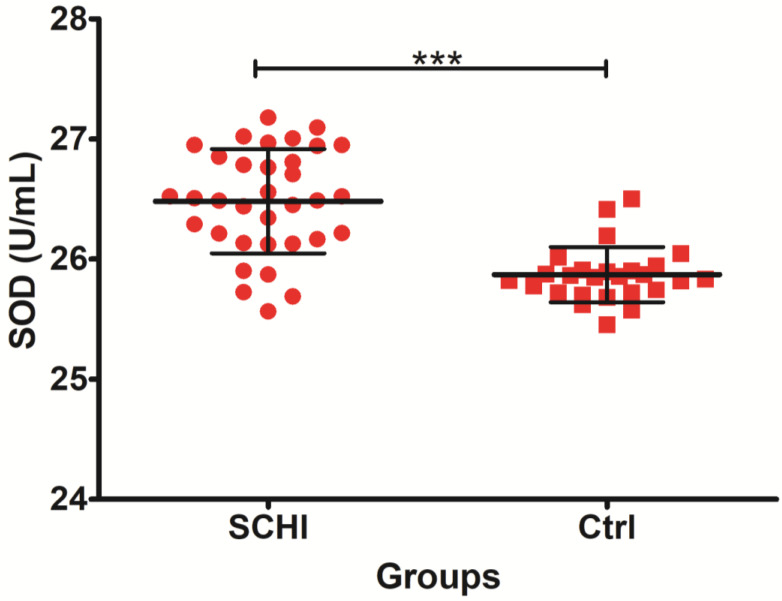
Levels of plasma superoxide dismutase SOD in schizophrenia group patients and healthy controls. *** indicates *p*-value < 0.0001.

The results presented in Figure 1 showed statistically significant (*p* < 0.00001) elevated activity of superoxide dismutase in patients with schizophrenia versus control group.

**Figure 2:**
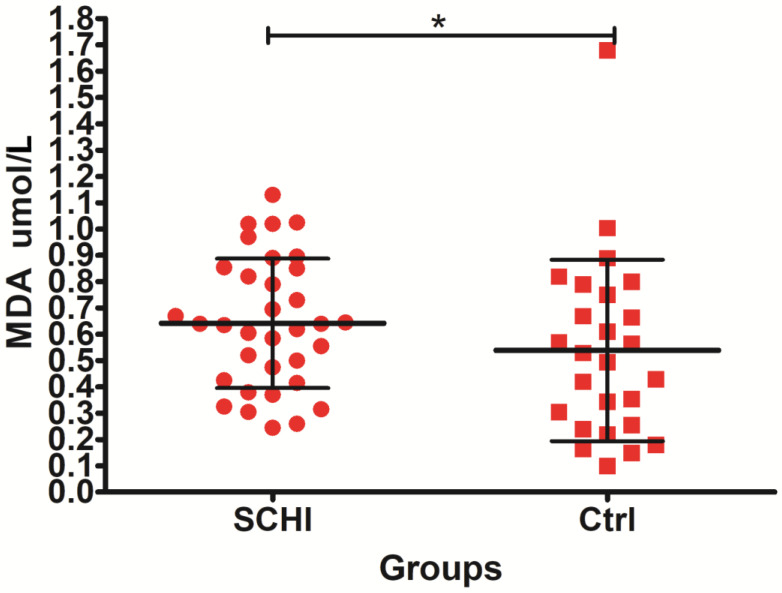
The level of malondialdehyde (MDA) concentration in the plasma of patients with schizophrenia compared to the control group. * indicates *p*-value < 0.05.

The conducted research presented in Figure 2 showed a statistically significant (*p* = 0.048) increased level of malondialdehyde in the plasma of patients with schizophrenia compared to the control group. 

**Figure 3:**
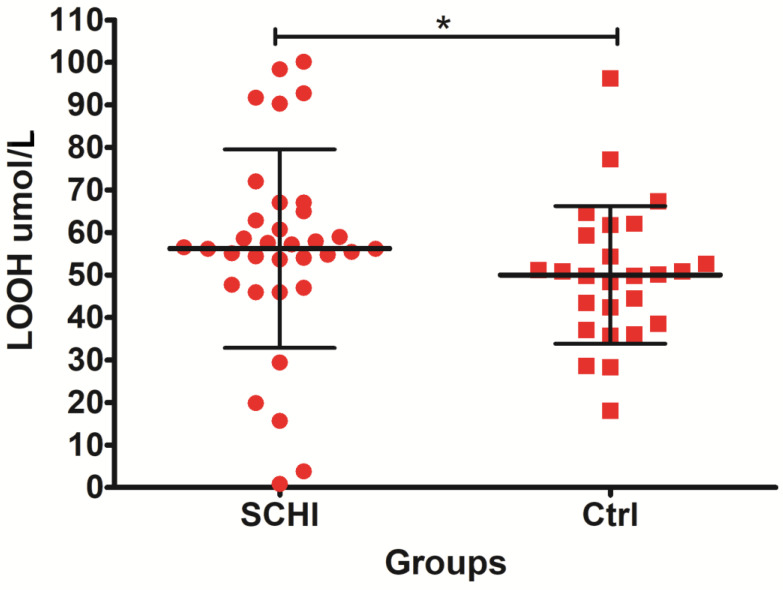
The level of lipid hydroperoxides (LOOH) concentration in the plasma of subjects with schizophrenia compared to the control group. * indicates *p*-value < 0.05.

Lipid hydroperoxide (LOOH) levels were statistically significantly higher (*p* = 0.034) in the first episode of patients with schizophrenia compared with the control group. The obtained results are presented in Figure 3.

The end-stage data analysis was to check the correlation between oxidative stress variables and the results of the PANSS scale. The mean total score on the PANSS was 97.04 ± 22.12, the result in the positive symptom subscale was 21.22 ± 8.16, and the negative symptom subscale was 26.25 ± 7.34. The results of the analysis are presented in Table 3.

A correlation between the oxidative stress variables, CRP and MDA, and the scores on the positive symptom subscale of the PANSS (PANSS P) was found in the present research. There was a positive correlation between MDA µmol/L and PANSS P and a positive correlation between CRP and PANSS P scale. No correlation was found for other variables such as SOD, CAT, LOOH, and FRAP.

Figures showing positive correlations are presented below.

**Figure 4:**
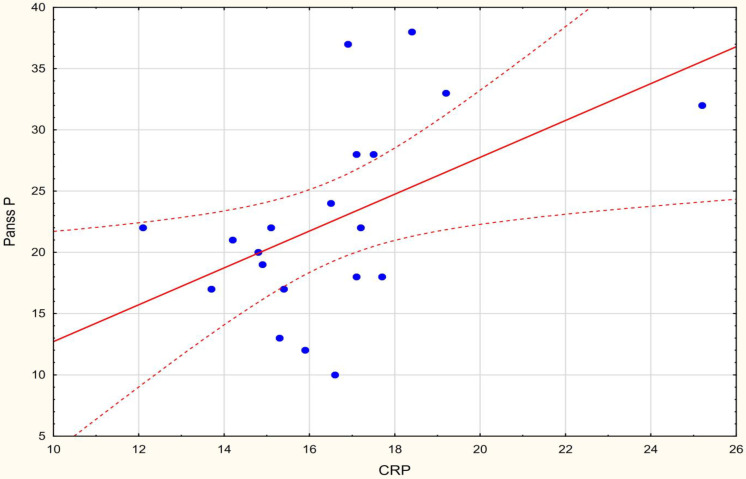
The correlation between C-reactive protein levels and positive-symptom scores.

C-reactive protein levels were positively correlated with positive-symptom scores in schizophrenia group patients (r Spearman = 0.491, *p* < 0.05). The results of the analysis are presented in Figure 4.

**Figure 5:**
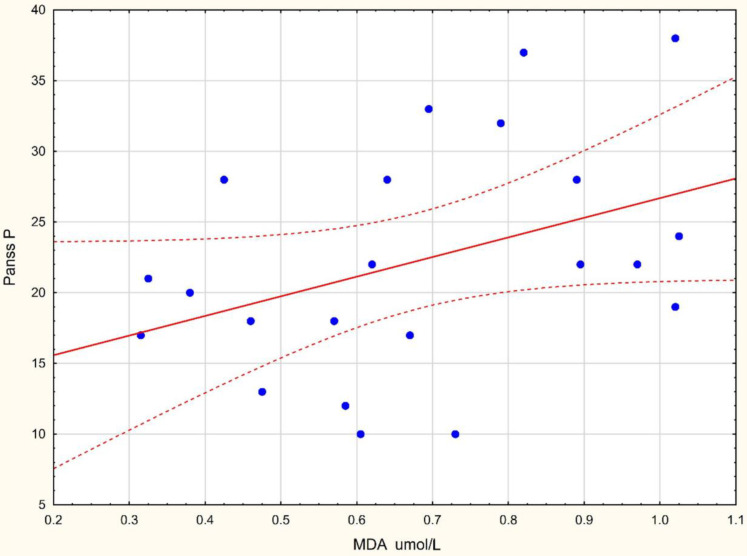
The correlation between MDA levels and positive-symptom scores.

Analysis showed that MDA level was positively correlated with positive-symptom stores (r Spearman = 0.439, *p <* 0.05). The results of the analysis are presented in Figure 5.

No correlation between the oxidative stress variables and CRP was found in the present research. The results are presented in Table 4. 

## 4. Discussion

Oxidative damage to the brain results from an increase in lipid peroxidation products in the cerebrospinal fluid and plasma and reduced polyunsaturated fatty acids levels in membranes of the brain and red blood cells. In response to the increase in reactive oxygen species, which leads to cell membrane damage caused by lipid peroxidation, the concentration of antioxidant enzymes may increase as a compensatory mechanism [5].

In our study, patients with their first episode of schizophrenia had significantly increased plasma SOD activity than controls. The other differences between CAT and FRAP were not statistically significant.

Superoxide dismutase is an important enzyme in the detoxification of superoxide radicals because these enzymes are the first substances produced in the majority of reactions that result in biological free radicals [11].

This result may be explained by compensatory mechanisms to counteract oxidative stress. The increased level of SOD may be associated with a hyperdopaminergic state in the nucleus accumbent, caudate nucleus, and amygdala, which leads to increased superoxide and H_2_O_2_ radical production [12].

In addition, studies on animal models of schizophrenia have shown increases in SOD activity, in conjunction with higher levels of lipid peroxidation in the prefrontal cortex [13].

In research by Ruiz-Litago et al., it was observed that the plasmatic levels of SOD in first-episode schizophrenia patients decreased significantly after one month and remained at a low level even after the 6-month follow-up period compared to the baseline measurement, which indicates an altered antioxidant defense at the onset of psychosis [14].

Other data have shown that erythrocyte SOD activity was increased in the early phase of schizophrenia, but its level depended on medical treatment. Administration of first-generation antipsychotics was associated with elevated SOD enzyme activity in all studied groups of patients [15,16,17]. In the study by Dietrich-Muszalska et al., the effect of a 4-week treatment with antipsychotics on the total antioxidant capacity of the plasma was assessed. The results showed that atypical antipsychotics were effective in antioxidant activity by reducing lipid peroxidation and increasing total plasma antioxidant activity [8].

If the compensatory mechanisms related to the increase in antioxidants are insufficient, the increase in ROS in cellular metabolism causes peroxidation of membrane lipids, proteins, and DNA, eventually leading to cell damage [18].

Present research conducted on patients in the first episode of schizophrenia showed that the level of MDA was higher than in the control group. Furthermore, elevated lipid oxidative damage, measured as increased levels of lipid hydroperoxides (LOOHs) in patients compared with healthy controls, may cause interference with the permeability and integrity of cell membranes in the brain.

There are literature reports indicating that an acute psychotic episode can be linked to low-grade systemic inflammation, evidenced by increased peripheral blood concentrations of cytokines and other markers of inflammation [19].

Our research indicates a positive correlation between CRP and PANSS P scale and also MDA and PANSS P scale. This may be an early indicator of ongoing low-grade inflammation. No correlation was found for other variables such as SOD, CAT, LOOH, and FRAP.

Some studies confirmed that the correlation between an inflammatory marker (CRP) and oxidative stress may suggest a link between inflammation and cellular stress responses [20,21,22]. Recent data indicate an increase in inflammatory markers in the peripheral blood of patients with schizophrenia during acute psychotic exacerbations, which suggests that immunological changes may affect the clinical condition of patients at the onset of the disease [23]. 

Various psychiatric disorders, including major depression, bipolar disorder, and schizophrenia, have been shown to be associated with increased levels of peripheral inflammatory markers. There is also evidence suggesting similar changes in the levels of CSF inflammatory markers in patients with schizophrenia compared to healthy controls [24]. Some of the studies revealed elevated levels of CRP in the group of patients with acute psychosis, which may be explained by the influence of stress associated with acute psychosis on the stimulation of the inflammatory response [24]. 

The inflammatory response leads to an increase in reactive oxygen species generation by endothelial cells and, as a result, to disruption of the blood–brain barrier (BBB) permeability. Furthermore, to the disruption of BBB and placental barrier permeability, cytokines in the brain regulate the expression of major histocompatibility complex I, coordinating synaptic pruning [25]; this was also confirmed by our previous studies [1]. Our results suggest significant relationships between the increased expression of apoptosis genes (*Bcl2*, *Birc6*, *Bax*, *Casp3*, and *Casp9*) in peripheral blood lymphocytes and immune system dysregulation in patients with schizophrenia.

It can therefore be assumed that the first important factor in activating the immune-inflammatory pathways is the activation of microglia and immuno-inflammatory processes caused by prenatal viral or bacterial infections. Subsequently, childhood infections can activate the immune system and promote the release of cytokines later in life with some stimulus. Another inflammatory factor activating the immune response is induced by a subsequent autoimmune response or life stress during adolescence [18].

Because, to date, there is no reliable biological marker for diagnostic schizophrenia, nor the possibility of preventing this condition, there is a need to identify specific indicators for diagnosis in patients with this disease.

Our research shows that low-grade inflammation may be a clinically useful feature in the prevention of schizophrenia, as it is associated with increased plasma levels of superoxide dismutase and malondialdehyde as indicators of low-level ongoing inflammation, particularly in children or adolescents.

Some studies reported that a low or normal level of pro-inflammatory cytokines in childhood is a possible protective factor [26], whereas the increased level of TNF-α in mothers with infectious disease in the third trimester of pregnancy leads to an eight-fold increase in the risk of psychotic disorder in the children as an adult [16].

## 5. Conclusions

The elevated level of SOD in patients with first-episode schizophrenia can be explained by compensatory mechanisms to counteract oxidative stress.

Malondialdehyde can be used as a simple biomarker of low-grade systemic inflammation associated with oxidative stress. MDA was proved to be a useful tool for identifying those at increased risk of the disease who may benefit most from early interventions.

A positive correlation between CRP and PANSS P scale and MDA and PANSS P scale may indicate a significant relationship between the development of low-grade inflammation and cell damage associated with oxidative stress in the development of the first symptoms of schizophrenia.

Due to the small number of patients, there is a need for further research on a larger group of patients with schizophrenia to confirm obtained results and continue other clinical observations.

## Figures and Tables

**Table 1:** The descriptive statistics of oxidative stress variables for the control group and patients with schizophrenia in plasma samples.

Variables	Group = Control					Group = SCHI				
	N	M	Me	SD	SEM	N	M	Me	SD	SEM
SOD (U/mL)	26	25.87	25.86	0.23	0.045	34	26.48	26.50	0.43	0.074
CAT U/L	26	49,778.96	48,505.75	23,232.02	4556.174	34	51,926.98	48,505.75	38,422.30	6589.370
LOOH µmol/L	26	50.01	50.00	16.21	3.180	34	57.65	56.34	20.61	3.534
MDA µmol/L	26	0.54	0.51	0.34	0.068	34	0.66	0.64	0.23	0.040
FRAP µmol/L	26	16.63	17.14	7.98	1.566	34	18.86	19.30	7.22	1.238
CRP mg/L	26	4.85	4.85	0.55	0.11	29 *	16.26	16.40	2.38	0.442

N, number of patients; M, mean; Me, median; SD, standard deviation; SEM, standard error of mean; SCHI, patients with schizophrenia; LOOH, lipid hydroperoxides; MDA, malondialdehyde; SOD, superoxide dismutase; GSH, glutathione; FRAP, Ferric Reducing Ability of Plasma; CRP, C-reactive protein; * CRP in the SCHI group was measured for 29 patients.

**Table 2:** Oxidative stress variables of patients versus control subjects.

U Mann-Witney Test	Sum of Rang Ctrl	Sum of Rang SCHI	U	Z	*p*
SOD (U/mL)	460.00	1370.00	109.00	−4.96757	0.000001
CAT U/L	829.50	1000.50	405.50	0.54449	0.586
LOOH µmol/L	660.50	1169.50	309.50	−1.97658	0.048
MDA µmol/L	651.50	1178.50	300.50	−2.11084	0.034
FRAP µmol/L	692.00	1138.00	341.00	−1.50668	0.131
CRP mg/L	1160.00	325.00	139.00	6.288	0.000001

**Table 3:** The results of the analysis of the correlation between the studied variables and the results of the PANSS scale. PANSS total, Positive and Negative Syndrome Scale for Schizophrenia total score; PANSS-P, Positive Scale; PANSS-N, Negative Scale; PANSS-G, General Psychopathology Scale.

Variables	PANSS P	PANSS N	PANSS G	PANSS T
SOD (U/mL)	−0.189	−0.223	−0.155	−0.252
CAT U/L	−0.128	−0.079	−0.396	−0.240
LOOH µmol/L	−0.171	−0.168	−0.214	−0.254
MDA µmol/L	0.439 *	−0.353	0.2125	0.181
FRAP µmol/L	0.368	0.159	0.359	0.384
CRP mg/L	0.491 *	−0.173	0.184	0.319

Legend: * means *p* < 0.05.

**Table 4:** The results of the analysis of the correlation between the CRP and the oxidative stress variables (both in SCHI and control group).

Variables	Group	
	Ctrl	SCHI
SOD (U/mL)	−0.031	−0.172
CAT U/L	0.023	0.242
LOOH umol/L	0.164	−0.036
MDA umol/L	0.065	0.356
FRAP umol/L	−0.374	0.268

## Data Availability

The data presented in this study are available on request from the corresponding author.
